# Morphological and physiological impacts of salinity on colonial strains of the cyanobacteria *Microcystis aeruginosa*

**DOI:** 10.1002/mbo3.1367

**Published:** 2023-06-28

**Authors:** Myriam Bormans, Benjamin Legrand, Nicolas Waisbord, Enora Briand

**Affiliations:** ^1^ UMR CNRS 6553 ECOBIO University of Rennes Rennes Cedex France; ^2^ UMR CNRS 6118 Géosciences Rennes University of Rennes Rennes Cedex France; ^3^ IFREMER, PHYTOX Laboratoire GENALG Nantes France

**Keywords:** colony, cyanobacteria, mesohaline estuary, mucilage, salinity shock

## Abstract

In the context of global change and enhanced toxic cyanobacterial blooms, cyanobacterial transfer to estuaries is likely to increase in frequency and intensity and impact animal and human health. Therefore, it is important to evaluate the potential of their survival in estuaries. In particular, we tested if the colonial form generally observed in natural blooms enhanced the resistance to salinity shock compared to the unicellular form generally observed in isolated strains. We tested the impact of salinity on two colonial strains of *Microcystis aeruginosa*, producing different amounts of mucilage by combining classical batch methods with a novel microplate approach. We demonstrate that the collective organization of these pluricellular colonies improves their ability to cope with osmotic shock when compared to unicellular strains. The effect of a sudden high salinity increase (*S* ≥ 20) over 5 to 6 days had several impacts on the morphology of *M. aeruginosa* colonies. For both strains, we observed a gradual increase in colony size and a gradual decrease in intercellular spacing. For one strain, we also observed a decrease in cell diameter with an increase in mucilage extent. The pluricellular colonies formed by both strains could withstand higher salinities than unicellular strains studied previously. In particular, the strain producing more mucilage displayed a sustained autofluorescence even at *S* = 20, a limit that is higher than the most robust unicellular strain. These results imply survival and possible *M. aeruginosa* proliferation in mesohaline estuaries.

## Graphical abstract

High salinity affects the morphology and viability of colonial strains of the cyanobacteria *Microcystis aeruginosa* producing different amounts of mucilage. We demonstrate that the collective organization of colonies embedded in an extracellular polymeric substance improves their ability to cope with osmotic shock when compared to unicellular strains. These results suggest *Microcystis* survival and a potential proliferation in mesohaline estuaries after transfer from freshwater.

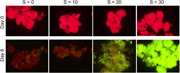

## 1 INTRODUCTION

Freshwater cyanobacterial blooms have been reported worldwide (Merel et al., 2013) and their proliferation together with their estuarine expansion has been increasing in recent years as a result of anthropogenic activities including eutrophication and climate warming (O'Neil et al., 2012; Paerl et al., 2018). These blooms represent environmental and human health hazards, as many species produce toxins harmful to animals and humans (Svirčev et al., 2017; Wood, 2016). The most common cyanotoxin detected in these blooms is microcystin produced by several species, including *Microcystis* (Harke et al., 2016). The transfer of cyanobacteria along the freshwater‐marine continuum has been observed worldwide (see Preece et al., 2017, for a review). Environmental studies show that *Microcystis aeruginosa* dominates these cyanobacterial transfers downstream as a result of its tolerance for high salinities (Tonk et al., 2007) in variable degrees among strains (Georges des Aulnois et al., 2019). At the single‐cell level, this resistance is mediated by the discharge and uptake of water in the cell (Hagemann, 2011; Kirsch et al., 2019). However, most environmental strains form pluricellular colonies packed within a layer of mucilage whose protective role remains unclear.

In their natural habitat, *Microcystis* colonies produce mucilage composed of exopolysaccharides, proteins, lipids, and nucleic acids to form scums (Liu et al., 2018; Reignier et al., 2023). Although the ecological impact of cyanobacteria colonies is still debated, some environmental conditions have been identified as proliferation factors: eutrophication of waters, a high concentration of calcium, magnesium, or lead, an increase in temperature, or the presence of other cyanobacteria and heterotrophic bacteria (Drugă et al., 2019; Sampognaro et al., 2020; Xiao et al., 2017). This colonial lifestyle is advantageous as it offers resistance to a host of biotic and abiotic threats and hence ensures better proliferation.

The impacts of salinity on natural colonies dominated by *M. aeruginosa* have been described in several publications including an enhanced production of mucilage, an increase in colony size, the reduction of intercellular space, the increase of cellular density within the colony (Kruk et al., 2017; Reignier et al., 2023; Sampognaro et al., 2020) and the decrease of growth rate (Kruk et al., 2017; Orr et al., 2004; Robson & Hamilton, 2003; Sellner et al., 1988).

Most strains of *Microcystis* that have been isolated from the environment will stop forming colonies when cultivated in the ideal growth conditions of a laboratory, where they usually adopt a unicellular lifestyle. Alongside other microbial biofilms, the colonies they form in their natural habitat are interpreted as a response to environmental stresses. Some isolated strains do retain the ability to produce mucilage and keep a colonial form in the early stages after isolation, but they produce less mucilage than natural colonies, and that ability is often lost as the isolated strains get older (Piccini et al., 2022; Wang et al., 2011). Because of these experimental limitations, the physiological responses of isolated strains of *M. aeruginosa* facing environmental stresses remain elusive. Particularly, the impact of salinity upon colonial forms of *M. aeruginosa* isolated strains is not known. This study focuses on the morphological and physiological responses of two isolated colonial strains of *M. aeruginosa* exposed to different salt concentrations mimicking the different phases of a land–sea transfer. Two complementary experimental approaches are used here: common batch cultures and a novel approach with a purposely built microplate to follow the time evolution of individual colonies.

## 2 MATERIALS AND METHODS

### 2.1 Biological material and culture conditions

Two isolated colonial *M. aeruginosa* strains were used for the experiments. Both strains were provided by the Paris Museum Collection (PMC). They were nonaxenic cultures but the process of isolation and rinsing together with the mineral culture medium ensured that the amount of bacteria was very limited. The strains were transported in sterile containers and manipulated under a PSM to minimize bacterial contamination.

**Figure 1:**
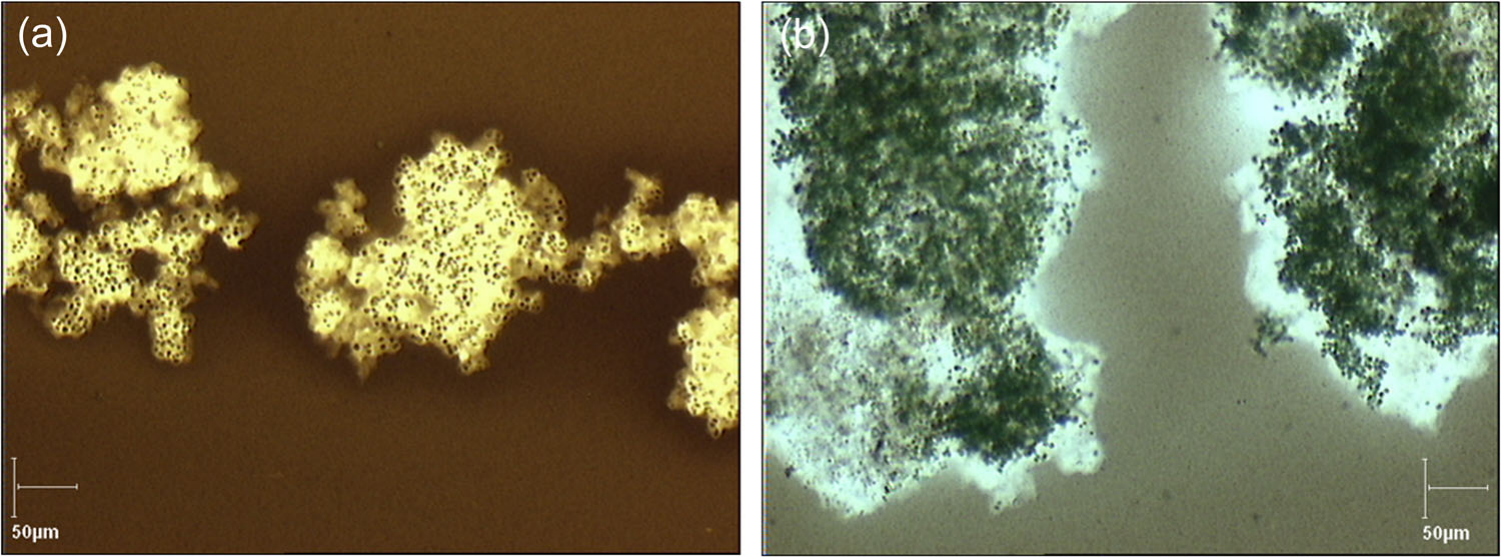
(a) *Microcystis aeruginosa* Paris Museum Collection (PMC) 1262.20 colonies and (b) *M. aeruginosa* PMC 1323.21 stained with Chinese ink to visualize the mucilage and observed with an optical microscope.

The first strain was *M. aeruginosa* PMC 1262.20 originating from Saintes Maries de la Mer in France and isolated in 2020. When grown in the control medium of BG11, these colonies had a diameter ranging from 100 to 200 µm and the cells had a diameter of 4 µm. The mucilage was thin and barely extended beyond the cells (Figure 1a). The colonies were sinking to the bottom of the culture medium.

The second isolated strain was *M. aeruginosa* PMC 1323.21 originating from Pannecière reservoir in France and isolated in 2021. The colonies of this strain in the control medium of BG11 were much larger than the previous one as they extended up to 2 mm in diameter. The cells had a similar diameter of 3.5 µm. The mucilage was thicker than the previous strain and extended further beyond the cells (Figure 1b) and the colonies floated to the surface of the culture medium. This strain was chosen to test if its resistance to a salinity increase was enhanced by the larger mucilage extent.

Both strains were cultured in BG11 medium at 22°C and a light intensity of 40 µE/m^2^/s with a photoperiod of 14/10 h. The cultures were regularly monitored by epifluorescence microscopy for bacterial contamination.

### 2.2 Staining protocols

Different staining protocols were used during the experiments to observe the physiological and morphological effects of salinity on the *M. aeruginosa* strains.

Chinese ink was used to show the extent of the mucilage as it is impermeable to it (mostly because the ink is composed of various particles and resins). The mucilage was then visualized as shown in Figure 1. A drop of classic Chinese ink on the sample before putting a cover slide was enough to visualize the mucilage. Measurements were achieved by the combination of a Sony digital camera mounted on an Olympus BX50 optical microscope, ×10, ×20, and ×60 objectives, and the Pegase imaging software.

The viability of the cells within the colonies was tested with SYTOX® Green Nucleic Acid stain (Molecular Probes; Invitrogen) for visualization of cyanobacterial cells with permeable membranes. The use of SYTOX® Green is based on the exclusion of the dye by cells with structurally integral membranes. The fluorophore penetrates damaged cells and exhibits the green fluorescence of nucleoids. We used an Olympus BX41 epifluorescence microscope equipped with a cool Led PE‐300 white light, with an excitation filter at 460–490 nm and an emission filter at 520 nm. After different concentration trials, the final concentration of SYTOX® Green retained was 1 µM as suggested by Tashyreva et al. (2013).

### 2.3 Batch experiments

For these experiments, colonies of both strains were grown in BG11 medium in Erlenmeyer flasks and kept in an exponentially growing phase. On Day 0, triplicates of 7 mL of BG11 with the addition of NaCl to reach the required salinities of *S* = 0, 10, and 30 for strain 1262.20 and of *S* = 0, 10, 20, and 30 for strain 1323.21 were transferred to 15 mL Falcon tubes. One milliliter of culture with an initial concentration of 8000 colonies/mL was centrifuged and the cell pellet was added to the culture media, corresponding to ~1200 colonies/mL. The batch cultures were stored in a growing chamber at 22°C and under a light intensity of 40 μE/m^2^/s with a photoperiod of 14/10 h.

The cellular viability and the volume of colonies were measured on the initial culture at Day 0, and in the different modalities on Days 1, 3, 5, and 7 for strain 1262.20 and on Day 6 for strain 1323.21. Colonies were fixed with a solution of 1% lugol for the measure of volume and cell counts performed on the next day (Meriluoto et al., 2017). The volume of colonies was inferred from the measure of the two horizontal axes and assumed the vertical axis was equivalent to the smaller of the two horizontal ones, as suggested by Sampognaro et al. (2020). A second batch experiment was performed in duplicates in 50 mL flasks over 3 days with the same salinities as in the first experiment to confirm the observations over that period. Cell counts and colony volumes were performed on an optical microscope in a Nageotte cell of 0.5 mm depth to avoid the spreading of the colonies by squeezing (Coudert et al., 2014; Meriluoto et al., 2017). The observations of the mucilage extent were achieved by adding one drop of Chinese ink between the slide and the slide cover to mark the colony contour. Cell size and intercellular space within colonies were measured on the Olympus BX50 optical microscope.

### 2.4 Microplate experiments

The microplate used during these experiments was made of polydimethylsiloxane (PDMS). This PDMS microplate lets O_2_ and CO_2_ gas exchange through the polymer. Hence, the microplate can be sealed to prevent evaporation and salt concentration in the wells. To create the microplate, 5 g of SYLGARD™ 184 Silicone Elastomer Curing Agent was added to 50 g of SYLGARD™ 184 Silicone Elastomer Base. The mixture was then placed under vacuum bell jar until all the bubbles disappeared from the mixture (usually after 20–30 min). The mixture was then poured onto a mold under vacuum bell jar for another 5 min and placed at 65°C to harden overnight. The microplate consisted of 18 wells (of a similar diameter to the typical 96 wells plate).

Each salinity concentration was prepared using a BG11 medium at 100 g/L of NaCl mixed with a classic BG11 medium to obtain culture media at *S* = 0, 10, 20, and 30. In each well, 180 µL of each salinity medium were added in triplicate to the microplate together with five colonies of either *M. aeruginosa* strain 1262.20 or *M. aeruginosa* PMC 1323.21. A positive control well was added with colonies exposed to 70% ethanol for 12 h before exposure to BG11 medium. This positive control gave the basal level of autofluorescence in the experiment where the autofluorescence is monitored as a proxy of chlorophyll photosynthesis.

The microplate filled with the different salt concentrations was placed under an inverted microscope (Leica DMi8) linked to a digital camera (Hamamatsu Orca Flash 4.0, SCOP PRO) with a time‐lapse software recording in live mode. Pictures of the wells were taken every 10 h for 5 or 6 days and then processed with ImageJ software to calculate the colony's autofluorescence intensity. The temperature was fixed at 20°C and the natural light had a photoperiod of 14/10 h. The excitation canal was at 640 nm and the emission filter was at wavelengths 662–738 nm.

### 2.5 Statistical tests

All of the data were evaluated on individual sampling dates by one‐way analysis of variance with Tukey's tests to identify differences between treatments and the control. Statistical tests and graphics have been performed using the “pairwise_tukeyhsd” method of the MultiComparaison function of the StatsModel python library. Significant differences are indicated by asterisks (*p < 0.05; **p < 0.01).

## 3 RESULTS AND DISCUSSION

### 3.1 Batch experiments

#### 3.1.1 Morphological changes

The aim of the batch cultures was to observe the effects of salinity on the morphology of the cells and colonies, and the extent of the mucilage at different salinities: *S* = 0, 10, 20, and 30 over several days of exposure. These salinities represent salinities measured during the transfer of *Microcystis* along the continuum from freshwater to estuary (Bormans et al., 2019). The change of volume of both colonies and cells with time was monitored in triplicates for each modality using an optical microscope. The extent of the mucilage was monitored through the extent of Chinese ink as reported by Piccini et al. (2022).

##### Strain 1262.20

**Figure 2:**
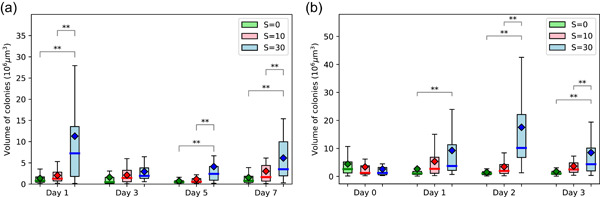
(a) Time evolution of colonies' volume (boxplot with the median as line and mean as diamant, *n* = 26) for strain 1262.20 as a function of salinity. Statistical differences were tested on each sampling day of the experiment (**p < 0.01). (b) Time evolution of colonies' volume (boxplot with the median as line and mean as diamant, *n* = 40) for strain 1262.20 as a function of salinity. Statistical differences were tested on each sampling day of the experiment (**p < 0.01).

We monitored the volume of colonies, the spacing between cells, and the size of cells. The volume of colonies varied both with salinity and with time (Figure 2a). The largest colonies were observed at *S* = 30. The statistically significant increase in volume observed after 1 day of exposition to high salinity (*S* = 30) was confirmed in a second experiment which lasted 3 days. This experiment also exhibited a significant increase in the volume of colonies on Days 1 and 2 at *S* = 30, while no significant changes were observed at *S* = 10 or in the control (*S* = 0) (Figure 2b). The large extent of the boxplots at *S* = 30 (Figure 2a,b) was associated with variabilities within each replicate rather than between replicates.

**Figure 3:**
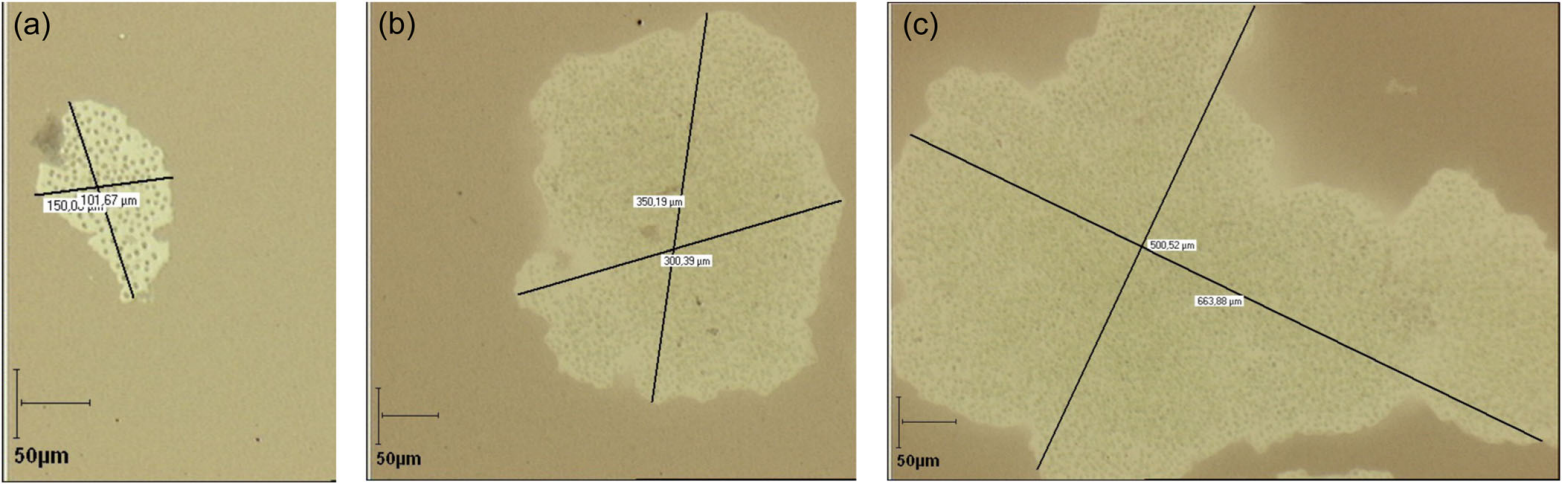
Representative examples of colony size on Day 2 at salinities (a) *S* = 0, (b) *S* = 10, (c) *S* = 30.

Figure 3 exhibits examples of colony size at different salinities on Day 2. The Chinese ink does not penetrate inside the colonies and at high salinity (*S* = 30) the colonies appear to result from the aggregation of smaller colonies rather than from cellular division. This increase of colony size with salinity was also reported on natural colonies by Sellner et al. (1988), Wang et al. (2022) and Sampognaro et al. (2020). Given the time scale, we assume that the colony size increase at high salinity results from aggregation and not cell division. High concentrations of calcium (Ca^+2^) (Wang et al., 2011), and other cations (Mg^+2^) present in natural waters (Drugă et al., 2019) have been reported to trigger aggregations of *Microcystis* colonies. Dervaux et al. (2015) have suggested that the aggregation occurred as a result of the flocculation between anionic extracellular polymeric substances and nutrient‐associated cations with a flocculation decrease with the ion valency, hence Ca^+2^ has higher flocculating power than Na^+^. This mechanism could explain the results obtained here at high salinity. The increase in colony size at high salinity has some ecological implications as it is likely to reduce predation and increase buoyancy leading to enhanced *Microcystis* proliferation.

**Figure 4:**
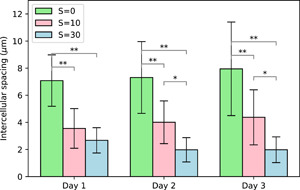
Time evolution of intercellular spacing within colonies as a function of salinity (mean ± SD, *n* = 15). Statistical differences between treatments were tested on each sampling day of the experiment (*p < 0.05; **p < 0.01).

The spacing between cells within the colonies also varied with salinity, with statistically significant smaller spacing at high salinity (Figure 4). This effect of smaller intercellular spacing within colonies with increasing salinity was also reported by Sampognaro et al. (2020) for natural colonies. They reported that intercellular spacing decreased with salinity shock up to *S* = 25.

**Figure 5:**
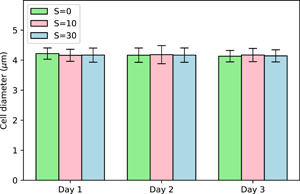
Time evolution of cell diameter as a function of salinity (mean ± SD, *n* = 20).

We also measured the cell size at different salinity exposures but we did not find any statistically significant variations either with time or with salinity (Figure 5). Some previous studies based on unicellular *Microcystis* indicated that cell size could be affected by salinity due to mechanisms associated with uptake and release of water (Georges des Aulnois et al., 2019; Hagemann, 2011) within few hours after exposure. The fact that we did not observe any changes on average cell size might be due to the timing of analyses as the first measurement was made after 24 h of exposure. Our results are similar to those of Tonk et al. (2007) who did not find any changes in cell size during the first 4 days after exposure while exposing a *M. aeruginosa* strain to a salt shock.

The extent of the mucilage beyond the colony boundaries did not change with time or with salinity, as observed with Chinese ink.

##### Strain 1323.21

**Figure 6:**
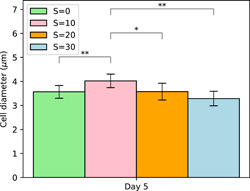
Measurements of cell diameter as a function of salinity at Day 5 (mean ± SD, *n* = 10). Statistical differences between treatments were tested (*p < 0.05; **p < 0.01).

Cell diameters were measured on several colonies as a function of salinity on Day 5. Our results show that cell size varied with increasing salinity displaying a statistically significant increase at low salinity followed by a steady decrease at higher salinities as shown in Figure 6. Cells at *S* = 30 are, however, most likely lysing or lysed as the cellular viability will show hereafter. The increase in cell size at low salinity (*S* = 10) is in line with the results of Wang et al. (2022). For natural colonies collected in situ and directly fixed in Lugol, the authors demonstrated by scanning an electronic microscope an increase in cell size of *Microcystis* from 2.42 to 2.71 µm at salinities respectively of *S* = 0 and 12. The decrease in cell size at higher salinity (*S* > 10) was also observed by Tonk et al. (2007) and Georges des Aulnois et al. (2019) on salt‐acclimated strains of *M. aeruginosa*. In particular, Tonk et al. (2007) suggested that osmoregulation was exceeded and that the cells were notwithstanding the high turgor pressure. Based on previous studies together with the present one, the effect of salinity on cell size seems to vary between strains, timing after exposure, and environmental conditions.

For this strain, at high salinities, it was not possible to accurately measure the intercellular spacing given the large density of cells in these colonies. This was interpreted as a qualitative indication that the intercellular spacing was very small, especially at higher salinities.

Chinese ink was used to test the extent of the mucilage on Day 5. Measurements performed with an optical microscope on colonies indicated a tendency for the maximum mucilage width beyond the cells to increase with increasing salinity, with values ranging from 43 μm at *S* = 0 to 105 μm at *S* = 30. These results are consistent with those observed in natural colonies by Sampognaro et al. (2020).

#### 3.1.2 Physiological changes

**Figure 7:**
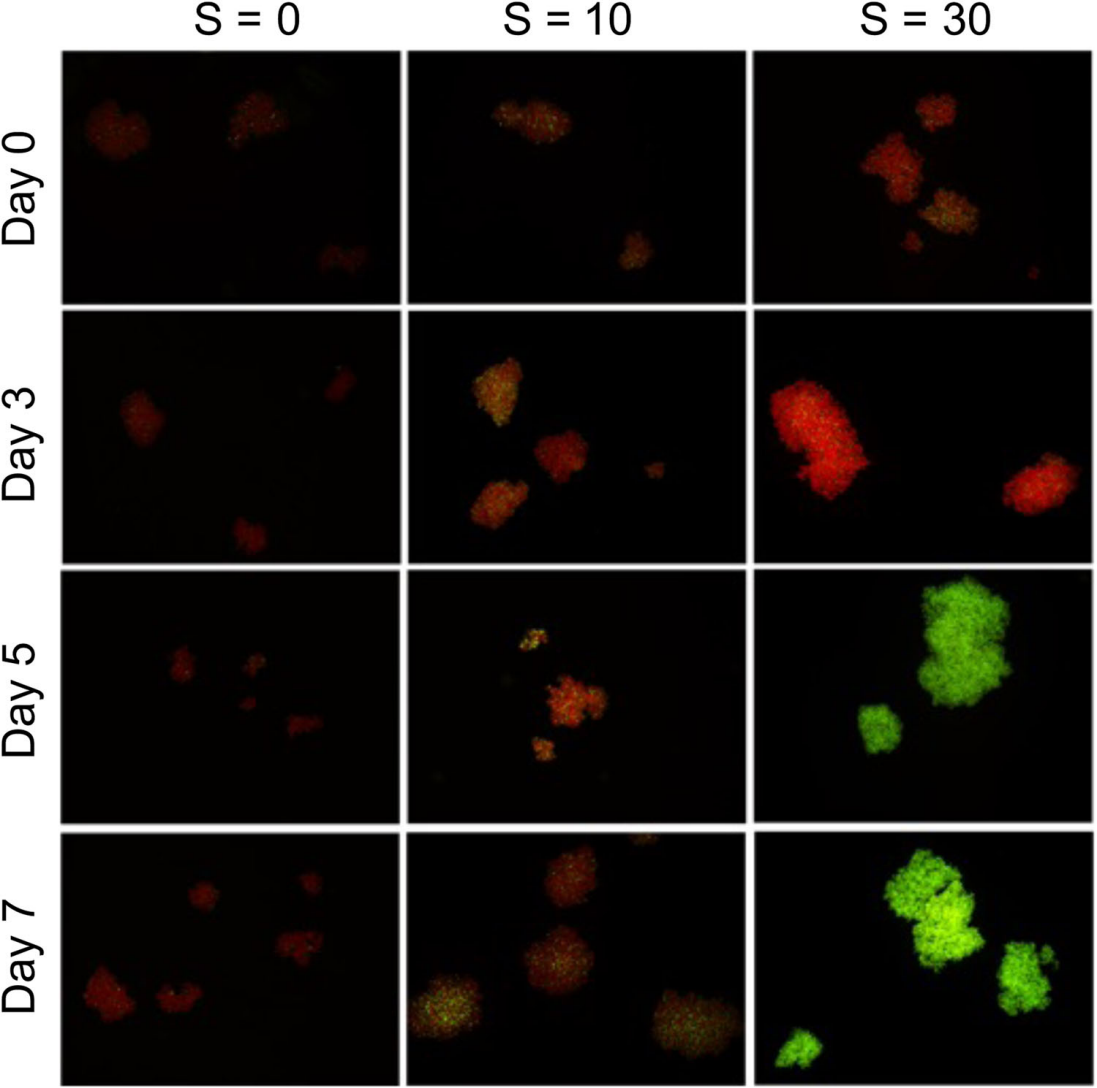
Time mosaics of colonies viability of strain 1262.20 as a function of salinity observed by epifluorescence microscopy using SYTOX Green fluorochrome.

For both strains, we monitored the cellular viability using epifluorescence microscopy and the SYTOX Green fluorochrome. Figure 7 displays the time mosaics for strain 1262.20 as a function of salinity. While the great majority of cells (marked red) were viable at *S* = 0, at *S* = 10 a significant proportion was marked green (dead cells), while all of the cells at *S* = 30 were marked green (as dead cells).

**Figure 8:**
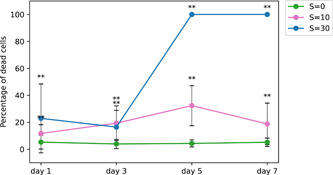
Percentage of dead cells as a function of time and salinity for strain 1262.20 (mean ± SD, *n* = 30). The statistical difference with the control was tested on each sampling day (*p < 0.05; **p < 0.01).

The percentage of dead cells as a function of time is presented in Figure 8. It is interesting to note that a nonnegligible variation in viability exposed to low salinity is observed in both Figures 7 and 8. Similar results were reported by Rosen et al. (2018) in a United States Geological Survey report. These authors showed that the cell viability decreased steadily with salinity increase up to *S* = 30. They also observed a nonnegligible variability within each condition, especially at high salinity with some colonies completely dead while others were still quite viable. While their results were based on natural colonies from the bloom of several *Microcystis* species, the dominant species was *M. aeruginosa*, and the shape of the colonies was definitively attributed to *M. aeruginosa*. Physiological variation among a population is logical as all the cells are not in the same phase of development even under cultured conditions.

Cellular viability using SYTOX Green fluorochrome by epifluorescence microscopy was also tested for strain 1323.21 on Day 0 and at the end of the experiment on Day 6 (*t* = 144 h).

**Figure 9:**
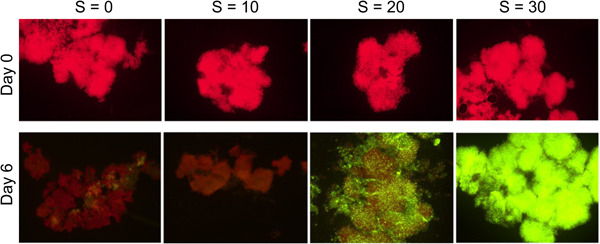
Cellular viability by epifluorescence microscopy at Days 0 and 6 for strain 1323.21 as a function of salinity.

On Day 6 (Figure 9), while the great majority of cells (marked red) were still viable at *S* = 0, at *S* = 10, a significant proportion (more than 50%) was marked green (dead cells), while all of the cells at *S* = 30 were marked green (as dead cells). An exact proportion of dead cells at *S* = 20 was not possible due to the high cell densities. However, this result indicates that some cells were still alive, suggesting a higher salinity threshold than unicellular strains which never grew beyond *S* = 15 (Black et al., 2011; Georges des Aulnois et al., 2020; Qiu et al., 2022).

### 3.2 Microplate experiments

The experiments using a purposely built microplate allowed us to isolate a few colonies per well and follow their evolution in time under a fixed condition, here salinity and ethanol without evaporation. Indeed, the sealed microplate made of PDMS let the transfer of gas (i.e., O_2_ and CO_2_), which is crucial for photosynthetic organisms. These experiments were run simultaneously with the batch experiments from the same initial culture to obtain complementary results.

#### 3.2.1 Strain 1262.20

**Figure 10:**
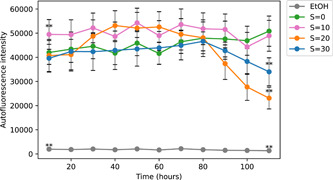
Time evolution of autofluorescence intensity for different salinities and ethanol exposures for strain Paris Museum Collection 1262.20 (mean ± SD, *n* = 10). Statistical differences with the control were tested on the first and last days of the experiment (**p < 0.01).

In this experiment which lasted 5 days, we used a microplate with three wells per modality. Different salinity exposures of *S* = 0, 10, 20, and 30 as well as exposure in ethanol used as a basal level of autofluorescence were tested. The results presented in Figure 10 show that even at *S* = 30 the level of autofluorescence was much higher than the basal level under the ethanol exposure. At zero and low salinity exposure (*S* = 10) the autofluorescence did not decrease with time over the 5 days while it decreased after 3 days at higher salinities (*S* = 20 and *S* = 30). After 5 days, the autofluorescence under *S* = 0 and *S* = 10 was much larger than for higher salinities of *S* = 20 and *S* = 30. These results indicate as expected some positive trend in chlorophyll fluorescence at zero salinity, and no trend at low salinity while high salinities of *S* = 20 and 30 have a considerable negative impact on the autofluorescence of colonies that are dead after 5 days as seen by the viability of cells (Figure 8). The level of autofluorescence in the salinities exposures remained higher than the basal level, suggesting a contribution from the phaeopigments to the autofluorescence signal (Le Rouzic et al., 1995).

#### 3.2.2 Strain 1323.21

We performed the same experiment with the microplate over 6 days with the *M. aeruginosa* colonial strain PMC 1323.21. We tested the autofluorescence after exposure at the same salinities *S* = 0, 10, 20, and 30 as well as after ethanol exposure.

**Figure 11:**
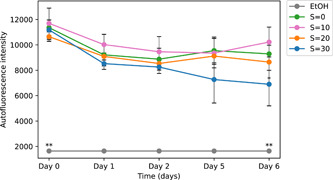
Time evolution of autofluorescence intensity for different salinities and ethanol exposures for strain Paris Museum Collection 1323.21 (mean ± SD, *n* = 5). Statistical differences with the control were tested on the first and last days of the experiment (*p < 0.05; **p < 0.01).

The results presented in Figure 11 show a concentration‐dependent response with lower autofluorescence at higher salinities. For all the salinity concentrations, there was a decrease in autofluorescence after 1 day. That decrease was also observed further in time at high salinity. This is consistent with the results observed for the first strain. These results indicate that after 6 days of exposure to salinities of up to *S*= 20 *Microcystis* colonies still maintain a high level of autofluorescence suggesting a higher salinity threshold than unicellular strains (Georges des Aulnois et al., 2020; Tonk et al., 2007). These results of autofluorescence confirm those of the cellular viability of the colonies and suggest a high salinity threshold for this strain producing larger colonies and more extended mucilage. It is interesting to note that similar to the results of the first strain (Figure 10), the autofluorescence at low salinity exposure is higher than that of the control. *Microcystis* (both as unicellular and colonial cells) exposed to low salinity have been reported to exhibit a better physiological state (Georges des Aulnois et al., 2020; Hagemann, 2011) and even growth rate (Robson & Hamilton, 2003) than the control. Again, all autofluorescence levels are significantly higher than the basal level for the first strain.

## 4 CONCLUSION

The effect of a sudden high salinity increase (*S* ≥ 20) over 5–6 days had several impacts on the morphology of *M. aeruginosa* colonies from two colonial strains. For both strains, we observed an increase in colony size, most likely from aggregation and a decrease in intercellular spacing. This increase in colony size has some ecological implications as it is likely to reduce predation and increase buoyancy leading to enhanced *Microcystis* proliferation. For one strain (the sinking one), we did not observe any change in cell size or mucilage extent while for the other strain (the floating strain) we observed a decrease in cell diameter together with a gradual increase in mucilage extent suggesting intraspecies variability also observed in unicellular *M. aeruginosa*.

The effect of salinity had also several impacts on the physiology of *Microcystis* colonies with a higher autofluorescence at low salinity compared to the control and similar cellular viability for both strains. High intrapopulation viability variability within one stress condition was, however, observed, suggesting different physiological statuses of individual colonies and even between cells within a colony. A higher cellular viability threshold than reported for unicellular strains was observed for both strains. Moreover, the strain producing more mucilage displayed a sustained autofluorescence even at *S* = 20 but this threshold still does not allow possible survival at marine salinity levels. The isolated strains displayed less mucilage than natural colonies, still, the mucilage seems to act as a protection against salinity stress. This result suggests that colonies embedded in mucilage are likely to survive in estuaries, as natural colonies potentially may have an even higher salinity tolerance threshold. This finding has important health implications for the organisms in estuaries likely to be exposed to these potentially toxic cyanobacteria. It also has major economic implications for the shellfish industries located in estuaries. Studying explicitly the mucilage composition of natural colonies under a salinity stress gradient would help in understanding more precisely their ecological role against osmotic stress and if the cyanobacteria can adapt their mucilage composition to overcome that particular stress.

The purposely built microplate allowed us to monitor the time evolution of individual colonies over several days. It would be interesting to test this microplate device on natural colonies and by extension use microfluidics to monitor the evolution of individual colonies under more complex environmental gradients.

## AUTHOR CONTRIBUTIONS

**Myriam Bormans**: Conceptualization (lead); formal analysis (equal); funding acquisition (lead); investigation (equal); methodology (equal); project administration (lead); supervision (lead); validation (lead); visualization (equal); writing—original draft (lead); writing—review and editing (equal). **Benjamin Legrand**: Formal analysis (lead); methodology (lead); visualization (equal); writing—original draft (equal); writing—review and editing (equal). **Nicolas Waisbord**: Conceptualization (equal); formal analysis (equal); methodology (equal); supervision (equal); writing—review and editing (equal). **Enora Briand**: Conceptualization (lead); formal analysis (equal); methodology (equal); supervision (equal); writing—review and editing (equal).

## CONFLICT OF INTEREST STATEMENT

The authors declare no conflict of interest.

## ETHICS STATEMENT

None required.

## Data Availability

The data are available at the following link: https://doi.org/10.48579/PRO/CM1FLM.

## References

[mbo31367-bib-0001] Black, K. , Yilmaz, M. , & Phlips, E. J. (2011). Growth and toxin production by *Microcystis aeruginosa* PCC 7806 (Kutzing) Lemmerman at elevated salt concentrations. Journal of Environmental Protection, 2(6), 669–674. 10.4236/jep.2011.26077

[mbo31367-bib-0002] Bormans, M. , Amzil, Z. , Mineaud, E. , Brient, L. , Savar, V. , Robert, E. , & Lance, E. (2019). Demonstrated transfer of cyanobacteria and cyanotoxins along a freshwater‐marine continuum in France. Harmful algae, 87, 101639. 31349891 10.1016/j.hal.2019.101639

[mbo31367-bib-0004] Coudert, L. , Rolland, D. , Blais, J.‐F. , Laurion, I. , & Mercier, G. (2014). *Etat de l'art en matière d'analyse des cyanobactéries et des cyanotoxines. Rapport final* (Rapport de recherche, R1475). INRS, Centre Eau Terre Environnement.

[mbo31367-bib-0005] Dervaux, J. , Mejean, A. , & Brunet, P. (2015). Irreversible collective migration of cyanobacteria in eutrophic conditions. PLoS One, 10(3), e0120906. 10.1371/journal.pone.0120906 25799424 PMC4370732

[mbo31367-bib-0006] Drugă, B. , Buda, D.‐M. , Szekeres, E. , Chiş, C. , Chiş, I. , & Sicora, C. (2019). The impact of cation concentration on *Microcystis* (cyanobacteria) scum formation. Scientific Reports, 9, 3017. 30816221 10.1038/s41598-019-39619-y PMC6395708

[mbo31367-bib-0007] Georges des Aulnois, M. , Réveillon, D. , Robert, E. , Caruana, A. , Briand, E. , Guljamow, A. , Dittmann, E. , Amzil, Z. , & Bormans, M. (2020). Salt shock responses of *Microcystis* revealed through physiological, transcript, and metabolomic analyses. Toxins, 12, 192. 32197406 10.3390/toxins12030192 PMC7150857

[mbo31367-bib-0008] Georges des Aulnois, M. , Roux, P. , Caruana, A. , Réveillon, D. , Briand, E. , Hervé, F. , Savar, V. , Bormans, M. , & Amzil, Z. (2019). Physiological and metabolic responses of freshwater and brackish‐water strains of *Microcystis aeruginosa* acclimated to a salinity gradient: Insight into salt tolerance. Applied and Environmental Microbiology, 85, e01614–19. 31444201 10.1128/AEM.01614-19 PMC6803299

[mbo31367-bib-0009] Hagemann, M. (2011). Molecular biology of cyanobacterial salt acclimation. FEMS Microbiology Reviews, 35, 87–123. 20618868 10.1111/j.1574-6976.2010.00234.x

[mbo31367-bib-0010] Harke, M. J. , Steffen, M. M. , Gobler, C. J. , Otten, T. G. , Wilhelm, S. W. , Wood, S. A. , & Paerl, H. W. (2016). A review of the global ecology, genomics, and biogeography of the toxic cyanobacterium, *Microcystis* spp. Harmful Algae, 54, 4–20. 28073480 10.1016/j.hal.2015.12.007

[mbo31367-bib-0011] Kirsch, F. , Klähn, S. , & Hagemann, M. (2019). Salt‐regulated accumulation of the compatible solutes sucrose and glucosylglycerol in cyanobacteria and its biotechnological potential. Frontiers in Microbiology, 10, 10. 31572343 10.3389/fmicb.2019.02139 PMC6753628

[mbo31367-bib-0012] Kruk, C. , Segura, A. M. , Nogueira, L. , Alcántara, I. , Calliari, D. , Martínez de la Escalera, G. , Carballo, C. , Cabrera, C. , Sarthou, F. , Scavone, P. , & Piccini, C. (2017). A multilevel trait based approach to the ecological performance of *Microcystis aeruginosa* complex from headwaters to the ocean. Harmful algae, 70, 23–36. 29169566 10.1016/j.hal.2017.10.004

[mbo31367-bib-0025] Le Rouzic, B. , Bertru, G. , & Brient, L. (1995). HPLC analysis of chlorophyll a breakdown‐products to interpret microalgae dynamics in a shallow bay. Hydrobiologia, 302, 71–80.

[mbo31367-bib-0013] Liu, L. , Huang, Q. , & Qin, B. (2018). Characteristics and roles of *Microcystis* extracellular polymeric substances (EPS) in cyanobacterial blooms: A short review. Journal of Freshwater Ecology, 33(1), 183–193. 10.1080/02705060.2017.1391722

[mbo31367-bib-0014] Merel, S. , Walker, D. , Chicana, R. , Snyder, S. , Baurès, E. , & Thomas, O. (2013). State of knowledge and concerns on cyanobacterial blooms and cyanotoxins. Environment International, 59, 303–327. 23892224 10.1016/j.envint.2013.06.013

[mbo31367-bib-0015] Meriluoto, J. , Spoof, L. , & Codd, G. A. (2017). Handbook of cyanobacterial monitoring and cyanotoxin analysis. John Wiley & Sons, Ltd.

[mbo31367-bib-0016] O'Neil, J. M. , Davis, T. W. , Burford, M. A. , & Gobler, C. J. (2012). The rise of harmful cyanobacteria blooms: The potential roles of eutrophication and climate change. Harmful algae, 14, 313–334.

[mbo31367-bib-0017] Orr, P. T. , Jones, G. J. , & Douglas, G. B. (2004). Response of cultured *Microcystis aeruginosa* from the Swan River, Australia, to elevated salt concentration and consequences for bloom and toxin management in estuaries. Marine and Freshwater Research, 55, 277.

[mbo31367-bib-0018] Paerl, H. W. , Otten, T. G. , & Kudela, R. (2018). Mitigating the expansion of harmful algal blooms across the freshwater‐to‐marine continuum. Environmental Science & Technology, 52, 5519–29. 29656639 10.1021/acs.est.7b05950

[mbo31367-bib-0019] Piccini, C. , Segura, A. M. , de la Escalera, G. M. , Croci, C. , & Kruk, C. (2022). New insight into colonies of *Microcystis* (cyanobacteria) as multi‐specific floating biofilms. EcoEvoRxiv. Preprint. 10.32942/osf.io/mqgr3

[mbo31367-bib-0020] Preece, E. P. , Hardy, F. J. , Moore, B. C. , & Bryan, M. (2017). A review of microcystin detections in estuarine and marine waters: Environmental implications and human health risk. Harmful algae, 61, 31–45.

[mbo31367-bib-0021] Qiu, Y. , Ma, Z. , Liu, X. , Zheng, R. , Xiao, Y. , & Wang, M. (2022). The detrimental effect of high salinity on the growth and microcystins contamination of *Microcystis aeruginosa* . Water, 14, 2871. 10.3390/w14182871

[mbo31367-bib-0022] Reignier, O. , Bormans, M. , Marchand, L. , Sinquin, C. , Amzil, Z. , Zykwinska, A. , & Briand, E. (2023). Production and composition of extracellular polymeric substances (EPS) by a unicellular strain and natural colonies of *Microcystis*: Impact of salinity and nutrient stress. Preprint. 10.21203/rs.3.rs-2818009/v1 PMC10667651 37697704

[mbo31367-bib-0023] Robson, B. J. , & Hamilton, D. P. (2003). Summer flow event induces a cyanobacterial bloom in a seasonal Western Australian estuary. Marine and Freshwater Research, 54, 139.

[mbo31367-bib-0024] Rosen, B. H. , Loftin, K. A. , Graham, J. L. , Stahlhut, K. N. , Riley, J. M. , Johnston, B. D. , & Senegal, S. (2018). *Understanding the effect of salinity tolerance on cyanobacteria associated with a harmful algal bloom in Lake Okeechobee Florida* (US Geological Survey Scientific Investigations Report 2018‐5092). p. 32. 10.3133/sir20185092

[mbo31367-bib-0026] Sampognaro, L. , Eirín, K. , Martínez de la Escalera, G. , Piccini, C. , Segura, A. , & Kruk, C. (2020). Experimental evidence on the effects of temperature and salinity in morphological traits of the *Microcystis aeruginosa* complex. Journal of Microbiological Methods, 175, 105971. 32544485 10.1016/j.mimet.2020.105971

[mbo31367-bib-0027] Sellner, K. G. , Lacouture, R. V. , & Parrish, C. R. (1988). Effects of increasing salinity on a cyanobacteria bloom in the potomac river estuary. Journal of Plankton Research, 10, 49–61.

[mbo31367-bib-0028] Svirčev, Z. , Drobac, D. , Tokodi, N. , Mijović, B. , Codd, G. A. , & Meriluoto, J. (2017). Toxicology of microcystins with reference to cases of human intoxications and epidemiological investigations of exposures to cyanobacteria and cyanotoxins. Archives of Toxicology, 91(2), 621–650. 28042640 10.1007/s00204-016-1921-6

[mbo31367-bib-0029] Tashyreva, D. , Elster, J. , & Billi, D. (2013). A novel staining protocol for multiparameter assessment of cell heterogeneity in phormidium populations (cyanobacteria) employing fluorescent dyes. PLoS One, 8, e55283. 23437052 10.1371/journal.pone.0055283 PMC3577823

[mbo31367-bib-0030] Tonk, L. , Bosch, K. , Visser, P. , & Huisman, J. (2007). Salt tolerance of the harmful cyanobacterium *Microcystis aeruginosa* . Aquatic Microbial Ecology, 46, 117–23.

[mbo31367-bib-0031] Wang, W. , Sheng, Y. , & Jiang, M. (2022). Physiological and metabolic responses of *Microcystis aeruginosa* to a salinity gradient. Environmental Science and Pollution Research, 29, 13226–13237. 34585353 10.1007/s11356-021-16590-8

[mbo31367-bib-0032] Wang, Y.‐W. , Zhao, J. , Li, J.‐H. , Li, S.‐S. , Zhang, L.‐H. , & Wu, M. (2011). Effects of calcium levels on colonial aggregation and buoyancy of *Microcystis aeruginosa* . Current Microbiology, 62, 679–683. 20872220 10.1007/s00284-010-9762-7

[mbo31367-bib-0033] Wood, R. (2016). Acute animal and human poisonings from cyanotoxin exposure—A review of the literature. Environment International, 91, 276–282. 26995270 10.1016/j.envint.2016.02.026

[mbo31367-bib-0034] Xiao, M. , Willis, A. , Burford, M. A. , & Li, M. (2017). Review: A meta‐analysis comparing cell‐division and cell adhesion in *Microcystis* colony formation. Harmful algae, 67, 85–91. 28755723 10.1016/j.hal.2017.06.007

